# Laparoscopy training of novices with complex curved instruments using 2D- and 3D-visualization

**DOI:** 10.1007/s00423-024-03297-w

**Published:** 2024-04-03

**Authors:** Johanna Hidding, Julian Bucher, Christian Heiliger, Dorian Andrade, Lukas Trupka, Matthias Halmen, Jens Werner, Konrad Karcz, Alexander Frank

**Affiliations:** 1 Department of Oral and Maxillofacial Plastic Surgery and Interdisciplinary Department of Oral Surgery and Implantology, University of Cologne, Cologne, Germany; 2 Department of General, Visceral and Transplantation Surgery, LMU University Hospital, Marchioninistr. 15, 81377 Munich, Germany

**Keywords:** Laparoscopy training, 3D Laparoscopy, Complex instruments, Visualization

## Abstract

**Purpose:**

Beside many advantages, disadvantages such as reduced degrees of freedom and poorer depth perception are still apparent in laparoscopic surgery. 3D visualization and the development of complex instruments are intended to counteract the disadvantages. We want to find out whether the use of complex instruments and 3D visualization has an influence on the performance of novices.

**Methods:**

48 medical students with no experience in laparoscopic surgery or simulator-based laparoscopy training were included. They were randomized in four groups according to a stratification assessment. During a structured training period they completed the FLS-Tasks “PEG Transfer”, “Pattern Cut” and “Intracorporeal Suture” and a transfer task based on these three. Two groups used conventional laparoscopic instruments with 3D or 2D visualization, two groups used complex curved instruments. The groups were compared in terms of their performance.

**Results:**

In 2D laparoscopy there was a better performance with straight instruments vs. curved instruments in PEG Transfer and Intracorporeal Suture. In the transfer task, fewer errors were made with straight instruments. In 2D vs. 3D laparoscopy when using complex curved instruments there was an advantage in Intracorporeal Suture and PEG Transfer for 3D visualization. Regarding the transfer exercise, a better performance was observed and fewer errors were made in 3D group.

**Conclusion:**

We could show that learning laparoscopic techniques with complex curved instruments is more difficult with standard 2D visualization and can be overcome using 3D optics. The use of curved instruments under 3D vision seems to be advantageous when working on more difficult tasks.

**Supplementary Information:**

The online version contains supplementary material available at 10.1007/s00423-024-03297-w.

## Introduction

Minimally invasive surgery (MIS) has become the gold standard of surgical therapy in many applications. This is, among others, caused by advantages over open surgery, such as less postoperative pain, lower infection rates, less intraoperative blood loss, shorter hospital stay, and faster convalescence [1]. However, minimally invasive techniques also come with disadvantages. These include impaired depth perception, which can lead to disorientation, increased tremor, limited field of view and unsteady camera movements and limited degrees of freedom [2–4]. In addition, many movement patterns are counterintuitive and consequently require significantly more training [5]. To improve training in MIS, various training modalities have been developed in the past with the goal of familiarizing surgeons with instruments and motion sequences in a controlled environment [6]. Among these, the most widely used curricula is the Fundamentals of Laparoscopic Surgery (FLS) program [7]. This is established as the standard for learning laparoscopic skills and simulates the skills necessary for laparoscopic surgery in five different tasks. Other ways to offset the disadvantages of conventional laparoscopy as presented could include 3D laparoscopy and the development of novel, complex instruments.

Unlike conventional 2D laparoscopy, 3D visualization impresses with improved depth perception [4]. Studies showed that with the use of 3D systems, exercises can be performed faster and more accurately than with conventional 2D systems [8]. The advantage of three-dimensional visualization over 2D vision has been demonstrated, especially in novices with no or minimal prior experience [9–11]. However, these systems are still not widely used, either because of high costs or the habit of experienced surgeons to 2D visualization [12, 13].

In order to overcome the limited degrees of freedom with the rigid and long instruments used in laparoscopy, developments are also moving in the direction of complex instruments. Probably the highest possible level of development at present in this respect are surgical robotic systems [14]. However, these are expensive and not available everywhere. Alternatively, articulated laparoscopic instruments bring advantages in cutting along complex structures and suturing at difficult angles, but also show a longer learning curve and lead to faster fatigue of the surgeon [15]. Also, the flexibility of these instruments may be perceived as a disadvantage [16]. Rigid, pre-bent instruments should overcome this issue. The instruments used primarily in single-incision laparoscopy should provide significantly more clearance for preparation, especially in parallel approaches [17]. The possible angulation, maybe combined with an additional rotation option of the instrument tip, should mimic the mobility of articulating instruments [18, 19].

Using three exercises from the FLS program (PEG Transfer, Pattern Cut and Intracorporeal Suture) and a transfer task developed by us based on them, this study will now test two questions on novice medical students inexperienced in laparoscopy: (1) What is the impact of using complex laparoscopy instruments compared to conventional laparoscopy instruments on novice laparoscopic performance? (2) Is 3D visualization beneficial in the use of complex laparoscopy instruments?

## Material and methods

### Participants

The study was reviewed and approved by local ethics committee (Ref. No.: 17–534). All participants gave written informed consent to participate in the study. 48 medical students in clinical semesters with no experience in minimally invasive surgery or simulator-based laparoscopy training (≤ 2 h) were included. Since the study has an observational character despite randomization, no power calculation was performed. The number of participants was determined in the basis of internal experience in similar studies. The recruitment was carried out among medical students at the Ludwig Maximilians University of Munich, Germany. In addition, all participants were tested for stereoscopic vision prior to study inclusion using the Lang II test (Lang II, Lang-Stereotest AG, Switzerland) [20]. Furthermore, a questionnaire was completed by each participant regarding demographics, experience in laparoscopy or laparoscopic training, and spatial orientation and stereoscopic vision.

### Setting and study design

**Fig. 1:**
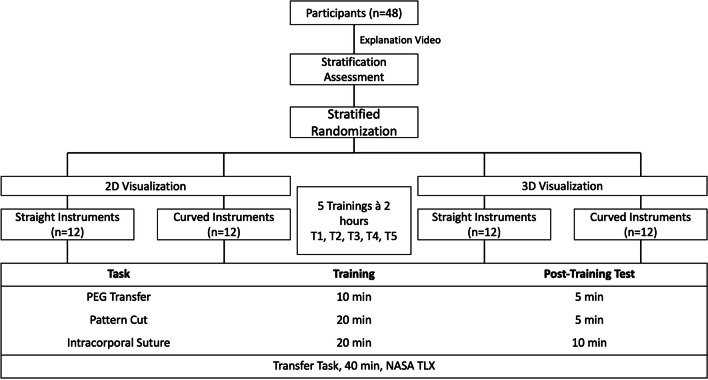
Study design. After watching an explanation video, the stratification assessment was conducted to be able to randomize the participant in four equal groups. Group I (*n* = 12): 2D visualization with straight instruments. Group II (*n* = 12): 2D visualization with one straight and one curved instrument. Group III (*n* = 12): 3D visualization with straight instruments. Group IV (*n* = 12): 3D visualization with one straight and one curved instrument. After Training and Test in PEG Transfer, Pattern Cut and Intracorporal Suture, the participance performed the transfer task. All participants executed 5 trainings within 2 h (T1-T5)

The study was performed in the minimal invasive surgery laboratory of the Department of General, Visceral and Transplantation Surgery of the University Hospital Munich. All participants received a video-based introduction to the Pattern Cut and Intracorporeal Suture tasks as well as an introduction to the handling of the laparoscopy instruments. Participants were then given 5 min to familiarize themselves with the setup and instruments. Afterwards, each participant performed the two tasks on a 2D box trainer. Based on the resulting performance score (Performance Score^Pattern^ + Performance Score^Suture^ = Performance score^Assessment^, see below), participants were stratified randomly into four groups with the goal of obtaining groups of equal strength (Fig. 1): Group I (*n* = 12) used straight laparoscopic instruments and Group II (*n* = 12) a pre-curved instrument each with 2D visualization, Group III (*n* = 12) used straight instruments and Group IV (*n* = 12) a pre-curved instrument each with 3D visualization. All tasks (for assessment and trial) were performed on a laparoscopy trainer according to Szabo-Berci-Sackier (Karl Storz GmbH & Co. KG, Tuttlingen, Germany) with fixed optics with 30° tilt. During the transfer task, participants had the opportunity to adjust the optics according to their requirements. All instruments and devices used in this study are listed in Table 1. For all groups the monitor was set up at a distance of 1.5 m at an angle of 180° to the subject. An additional monitor was placed aside to watch the tutorial videos. The participants in the 3D groups wore shutter glasses (Hama GmbH & Co. KG, Monheim, Germany).

**Table 1:** Material

	Manufacturer
Straight Instruments	
Needle driver	26,173 KL, KARL STORZ GmbH & Co. KG, Tuttlingen Germany
Grasping forceps	31,351 MD CLICKlinie, Kelly dissecting and grasping forceps; KARL STORZ GmbH & Co. KG, Tuttlingen, Germany
Scissor	31,351 MW CLICKline, serrated, curved, conical; KARL STORZ GmbH & Co. KG, Tuttlingen, Germany
Shaft	Length 30 cm, size 5 mm; KARL STORZ GmbH & Co. KG, Tuttlingen, Germany
Curved Instruments	
DuoRotate handgrip	1,152,261; Richard Wolf GmbH, Knittlingen, Germany
DuoRotate shaft	Length 38 cm, size 5.8 mm, 1,152,266; Richard Wolf GmbH, Knittlingen, Germany
DuoRotate grasping forceps	Maryland, size 5.8 mm, 1,152,319; Richard Wolf GmbH, Knittlingen, Germany
DuoRotate scissor	Metzenbaum, size 5.8 mm, 1,152,321; Richard Wolf GmbH, Knittlingen, Germany
2D Visualization	
Fiber optics	295 NB LOT VU61; KARL STORZ GmbH & Co. KG, Tuttlingen, Germany
Light source	201,331 01–1, cold light fountain, XENON 300 SCB; KARL STORZ GmbH & Co. KG, Tuttlingen, Germany
Camera	Telecam 20,212,030, PAL, SN YU859219-H; KARL STORZ GmbH & Co. KG, Tuttlingen, Germany
Optic	HOPKINS Forward-Oblique Telescope, 30° optic, 26,003 BA HOPKINS, SN121TR5; KARL STORZ GmbH & Co. KG, Tuttlingen, Germany
Monitor	WideViewTM HD; KARL STORZ GmbH & Co. KG, Tuttlingen, Germany
3D Visualization	
Fiber optics	80,655,030 Fusion Fiber, 5 mm GL 3 m, 1,214,622, Richard Wolf GmbH, Knittlingen, Germany
Light source	5,162,001, Endolight LED 1.3 76W, 1,100,221,268, Richard Wolf GmbH, Knittlingen, Germany
Camera	Endocam Epic 3DHD camera system, 5,531,001, 1,100,214,043; Richard Wolf GmbH, Knittlingen, Germany
Optic	8,934,632, 3D-Endoscope 30°, 10 mm NL 301 mm, 1,100,211,334; Richard Wolf GmbH, Knittlingen, Germany
Monitor	LMD-3251MT, LCD Monitor, 3,100,108; Richard Wolf GmbH, Knittlingen, Germany
Shutter glasses	109,804; HAMA GmbH & Co. KG, Monheim, Germany
Boxtrainer (all groups)	
Boxtrainer	SZABO-BERCI-SACKIER, 26,348; KARL STORZ GmbH & Co. KG, Tuttlingen, Germany

Instruments and material used within the study

Five training sessions of two hours each were planned for each participant, in which both training and the FLS test had to be completed in a predefined order. After a learning video, the sessions were completed as shown in Fig. 1. Within the given training time, the exercises could be repeated as many times as possible. This was followed by a 40 min transfer task (see below). All five training sessions were to be completed within 2 weeks with an interval of at least 24 h between two training sessions.

Participants in groups I and III used conventional straight laparoscopic instruments (Karl Storz GmbH & Co. KG, Tuttlingen, Germany). Participants in groups II and IV used curved DuoRotate instruments (Richard Wolf GmbH, Knittlingen, Germany) in addition to the conventional straight instruments (Table 1).

### Exercises and performance score

PEG Transfer: Participants were asked to pick up each of six objects in turn from a pegboard with the non-dominant hand, transfer it to the dominant hand, and place it back on the pegboard on the other side and vice versa [21]. The performance score was calculated as follows: *Performance Score* = *maximum time allowed (300 s)—time required (sec)—(10* × *number of errors)*. Any loss of the PEG was counted as an error. If the PEG fell out of the field of view, this was counted as two errors [22]. Participants in group I and III used two straight grasping forceps, participants in group II and IV used one straight and one curved grasping forceps (Table 1).

Pattern Cut: participants were asked to cut a circle with a diameter of 7.4 cm from a stretched cellular material [21]. The performance score was calculated as follows: *Performance score* = *maximum time allowed (300 s)—time required (sec)—(20* × *errors)*. Miscuts of more than 5 mm both inside and outside the mark were considered as errors [22]. Participants in group I and III used straight grasping forceps and straight scissors for this purpose, while participants in group II and IV used straight grasping forceps and curved scissors.

Intracorporeal Suture: A 3 cm long rubber band with a slit-shaped opening and two marked target points was fixed in the training box. Participants were asked to place a suture in the area of the target points and knot it. First, a double knot should be made, followed by two single knots [21]. The performance score was calculated as follows: *Performance score* = *maximum time allowed (600 s)—time required (sec)—(10* × *accuracy error)—(10* × *safety error)*. The accuracy error was the deviation in mm from the markings, and the safety error was a slip of the knot with one and a loosening of the knot with two error points [23]. Participants in group I and III used straight grasping forceps and a straight needle holder, participants in groups II and IV used curved grasping forceps and a straight needle holder.

**Fig. 2:**
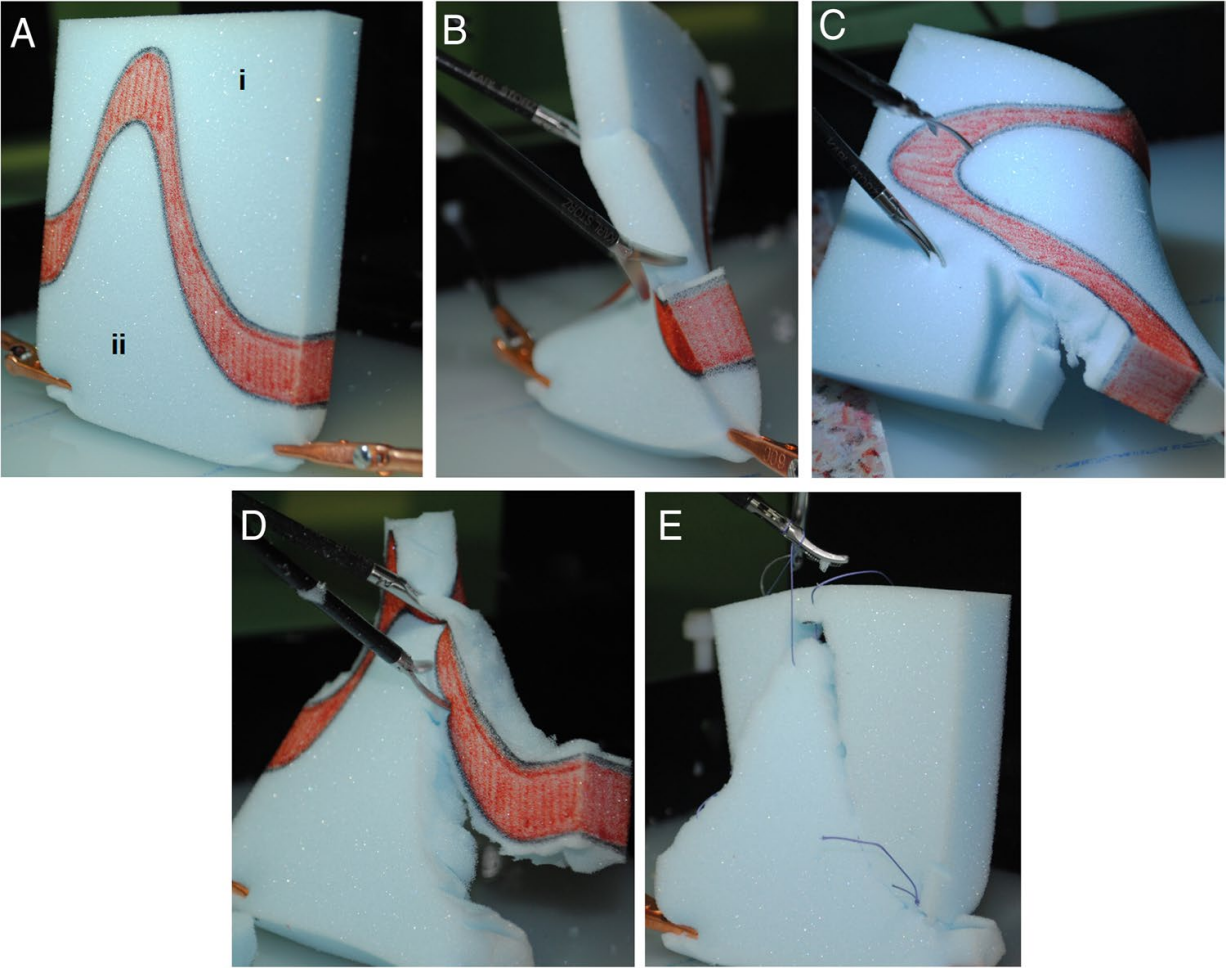
Transfer task. A foam piece was fixed upright in the box trainer (**A**). To cut out the red marked area the participant had to move the foam piece with a grasper (**B**-**D**). After the mold was cut out, the remaining pieces (i, ii) should be sewn together with three single sutures (**E**)

Transfer task (Fig. 2): The objective was to cut out a three-dimensional red marked shape along two 1 mm wide lines from a 10 × 12 × 1.5 cm piece of foam. The foam piece was fixed upright in the box trainer. In doing so, the participant had to move the foam piece with a grasper. Adjusting the camera or moving the camera to another trocar were allowed analogous to the real surgical situation. After the mold was cut out, it was to be set aside and the remaining pieces sewn together with three single sutures. The transfer task is a combination of the skills from the previous tasks. Due to its complexity, the maximum time was set at twice the sum of the three tasks. This results in a maximum time of 2400 s for the transfer task. The maximum time to complete the task was 2400 s. The performance score was calculated as followed: *Performance score* = *maximum time allowed (2400 s)—time required (sec)—(number of red rests x length of red rests in mm)—(0.5* × *number of miscuts x length of miscuts in mm) – [100x (red sections-1)]* + *[100* × *number of correct knots* + *50* × *number of correct sutures (only if no knots were made)].* Participants in group I and III used straight scissors and straight grasping forceps for cutting and straight needle holder and straight grasping forceps for sewing. Group II and IV participants used curved scissors and straight grasping forceps for cutting and straight needle holder and curved grasping forceps for sewing.

### Data collection and statistical analysis

Data collection was carried out during the test by the investigator. In addition to the time, the respective parameters were collected analogous to the FLS guidelines [21, 22]. For the transfer task, time was also recorded by the investigator. Errors were recorded analogous to the respective FLS task, which corresponded to the sub steps of the transfer task.

Statistical analysis was performed using SPSS version 25.0 (IBM SPSS Statistics, Armonk, New York, USA). The significance level was set at a *p* < 0.05.

## Results

### Demographic data

A total of 48 subjects were included in the study. They were recruited from clinical semesters with an average of 9.4 ± 2.1 semesters (5–13, according to German study regulations on medicine). Age was 25.8 ± 3.3 years (22–33 years) and 66.7% of participants were female (*n* = 32). The overall performance score for group stratification was -151.4 ± 146.9 (-420–206, -150.8). Overall, there were no differences in age, gender, semester of study, and initial performance score, as well as spatial orientation and 3D vision (Tables 2 and 3). None of the participants had prior experience in laparoscopic surgery or laparoscopic training and therefore met the inclusion criteria.

**Table 2:** Demographic data of participants after stratified randomization

	Overall (*n* = 48)	Group I (*n* = 12)	Group II (*n* = 12)	Group III (*n* = 12)	Group IV (*n* = 12)	*p*-value
Gender						
Female [%] (n)	66.7 (32)	58.3 (7)	83.3 (10)	66.7 (8)	58.3 (7)	0.522
Male [%] (n)	33,3 (16)	41.7 (5)	16.7 (2)	33.3 (4)	41.7 (5)	
Age [years] mean ± SD (range; median)	25.8 ± 3.3 (22–33; 24.5)	26.2 ± 3.4 (22–32; 25)	26.2 ± 3.6 (22–35; 25.5)	25.3 ± 3.8 (22–33; 24)	26.5 ± 4.3 (22–34; 24.5)	0.804
Semester, mean ± SD (range; median)	9.4 ± 2.1 (5–13; 9)	9.5 ± 1.3 (7–12; 9)	9.8 ± 2.5 (5–13; 10)	8.18 ± 1.9 (5–11; 9)	9 ± 1.5 (6–11; 9)	0.273
*P*-Score stratification assessment, mean ± SD (range; median)	-151.4 ± 146.9 (-420–205; -150.8)	-117.3 ± 162.4 (-400–179; -107.5)	-189.1 ± 139 (-420–84; -215)	-121.1 ± 150.1 (-390–205; -127.5)	-178.1 ± 118 (-400–3; -190.75)	Table 3

No difference was shown for gender, age, semester and performance score of the stratification assessment. *Group I* 2D visualization with straight instruments, *Group II* 2D visualization with curved instruments, *Group III* 3D visualization with straight instruments, *Group IV* 3D visualization with curved instruments, *P-Score* performance score of stratification assessment, *SD* standard deviation

**Table 3:** Comparison of different randomized groups according to the performance score of stratification assessment

Group	*p*-value
Group I vs. Group IV	0.667
Group I vs. Group II	0.920
Group I vs. Group III	0.944
Group IV vs. Group III	0.575
Group IV vs. Group II	0.779
Group III vs. Group II	0.841

There was no difference between the randomized groups. For man and standard deviation see Table 2. *Group I* 2D visualization with straight instruments, *Group II* 2D visualization with curved instruments, *Group III* 3D visualization with straight instruments, *Group IV* 3D visualization with curved instruments

### Conventional 2D laparoscopy with straight vs. complex curved instruments

**Fig. 3:**
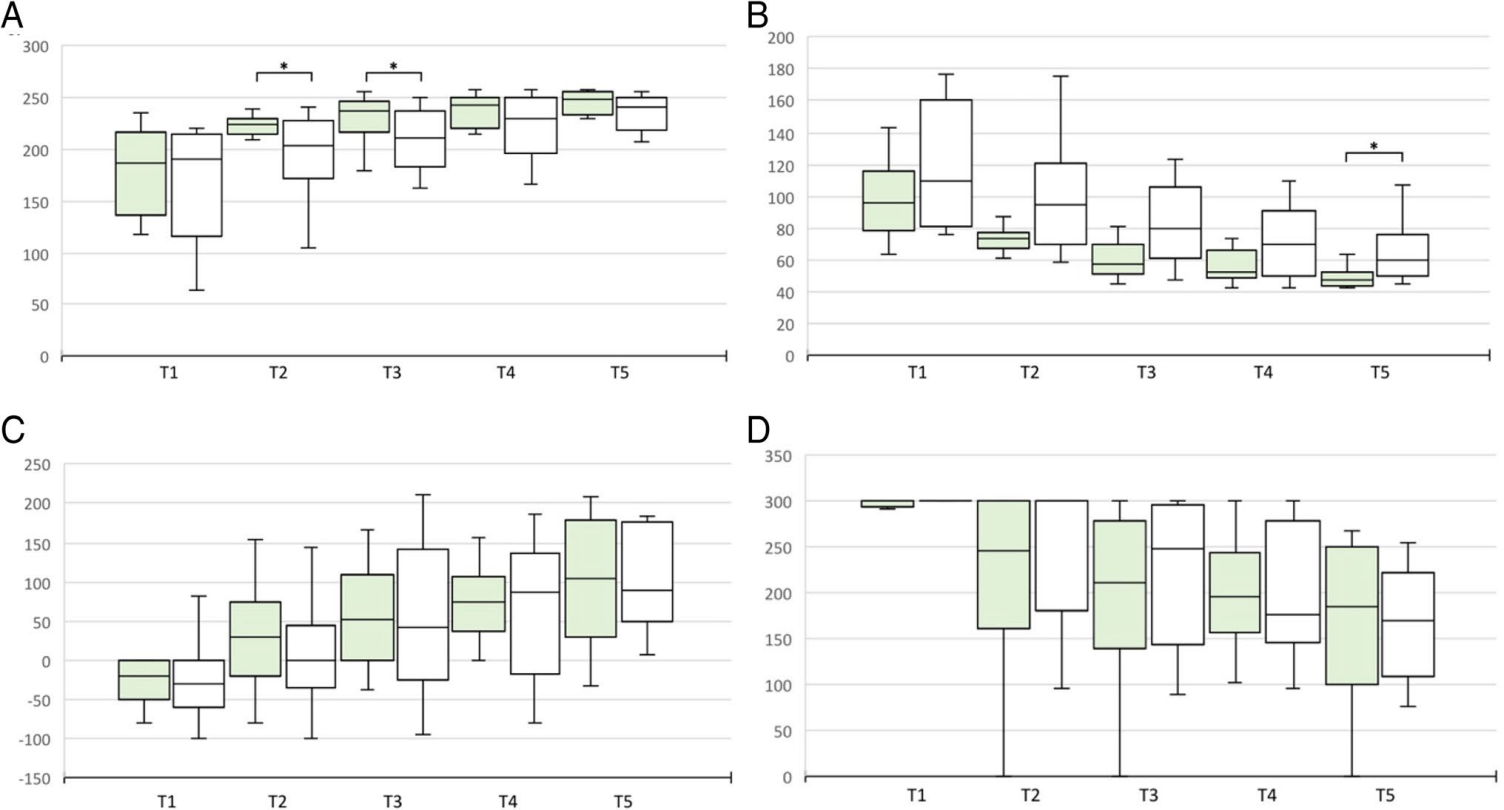
Performance score and time of PEG Transfer and Pattern Cut exercise in 2D visualization straight vs. curved instruments at T1-5. (**A**) Performance score PEG Transfer. **B** Time PEG Transfer. **C** Performance Score Pattern Cut. **D** Time Pattern Cut. Green: Straight instruments. White: Curved Instruments. *p < 0.05

**Fig. 4:**
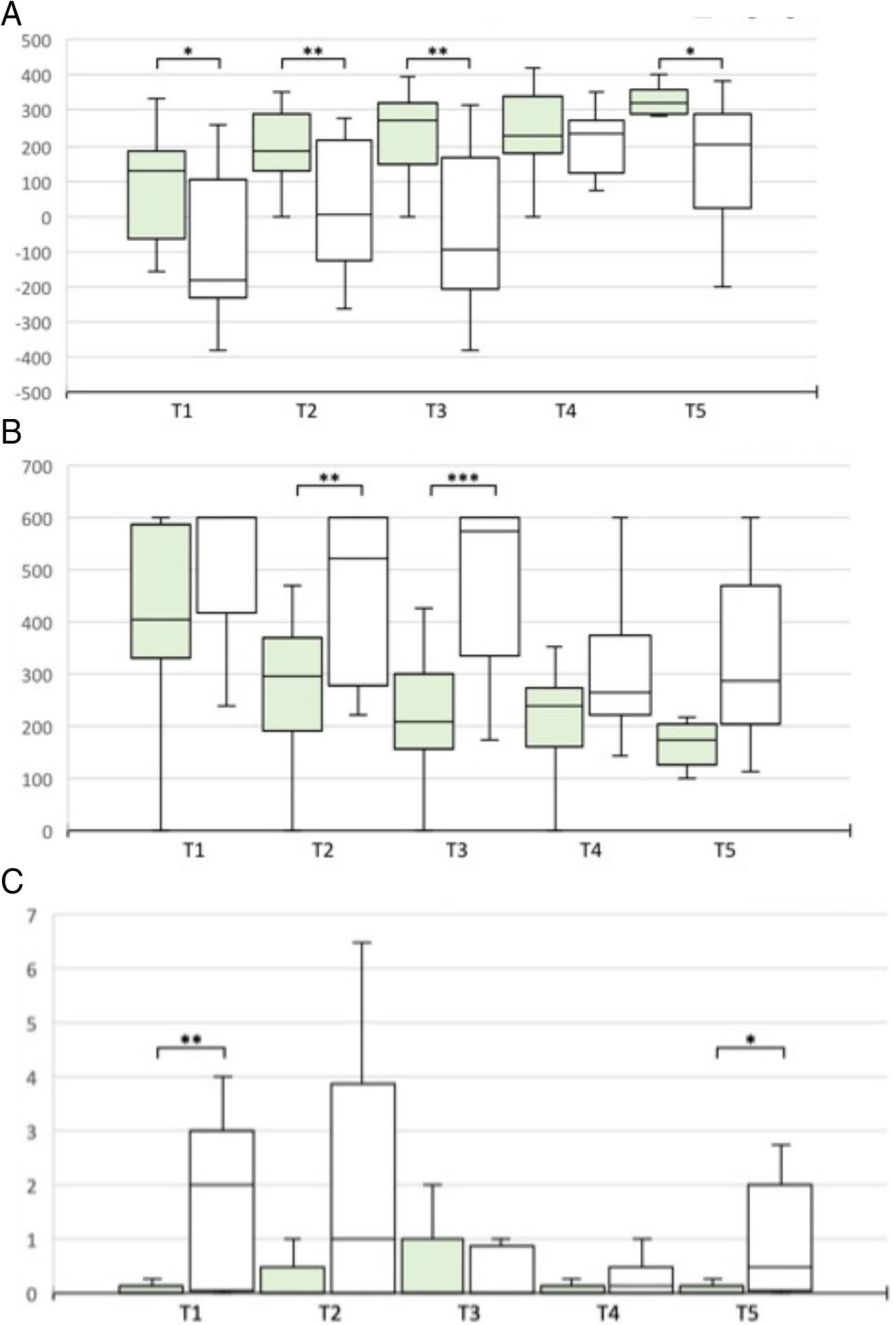
Performance score, time and errors of Intracorporeal Suture exercise in 2D visualization straight vs. curved instruments at T1-5. (**A**) Performance score. **B** Time. **C** Errors. Green: Straight instruments. White: Curved instruments. *p < 0.05; **p < 0.01; ***p < 0.001

For the performance score of the exercise PEG transfer, we could detect a significant difference at test time T2 and T3. The subjects in the group with straight instruments performed better (224.1 ± 8.6 vs. 190.1 ± 54.1, p = 0.024 and 233.4 ± 21.1 vs. 208.3 ± 28.6, p = 0.028). Even though there was no significant difference between the two groups on the last day, one could however see a tendency for better results in the straight instrument group (244.8 ± 16.6 vs. 232 ± 23.6, p = 0.06). This assumption is also consistent with the fact that at test time T5 the task was performed faster in the group of straight instruments (50.2 ± 7.2 s vs. 64.7 ± 17.7 s, p = 0.041) (Fig. 3). No significant differences could be found in the Pattern Cut exercise. Only for the number of errors at test time T5 a tendency for the advantage of straight instruments can be derived (0.58 ± 1.17 vs. 1.25 ± 0.97, p = 0.06). The Intracorporeal Suture exercise showed better performance in the straight instrument group at several test time points, including T5 (305 ± 100.5 vs. 147.3 ± 191.5, p = 0.012), as well as fewer errors (0.76 ± 2.09 vs. 0.97 ± 1.03, p = 0.033). Even though participants took less time to complete the task at test time T5, this difference was not significant (188.1 ± 82.7 vs. 329.8 ± 165.4, p = 0.087) (Fig. 4).

**Fig. 5:**
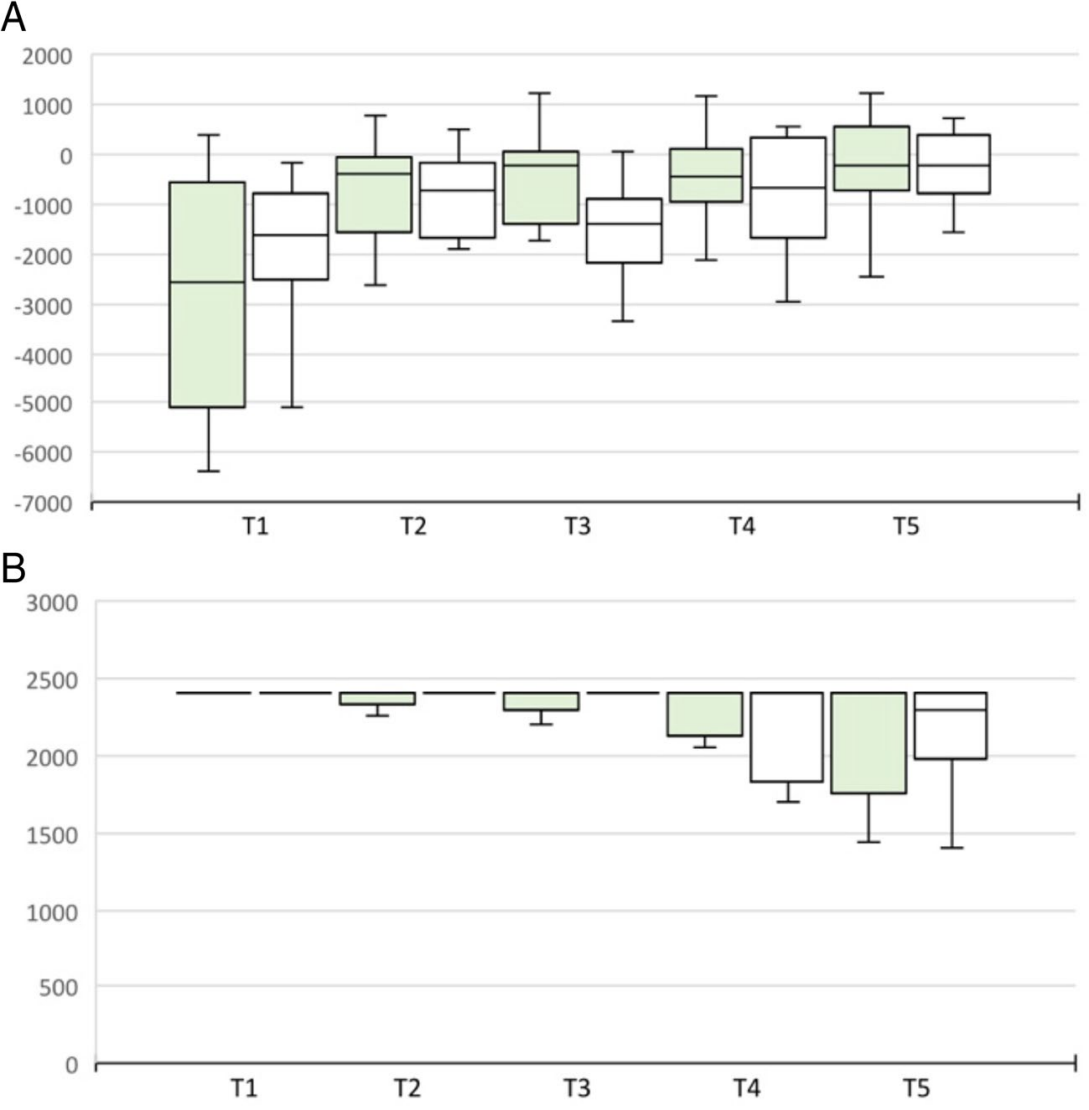
Performance score and time of Transfer Task in 2D visualization straight vs. curved instruments at T1-5. (**A**) Performance score. **B** Time (Time < 2400 s: T1 *n* = 3; T2 *n* = 9; T3 *n* = 5; T4 *n* = 17; T5 *n* = 25). Green: Straight instruments. White: Curved instruments

In the transfer task, there was no difference between the two groups in terms of performance and time. However, at test time T1 only three and at test time T5 only about half of the novices solved the task in the given time. Since the time was only one aspect of the calculation of the performance (see above), the errors were also considered independently. Here it was noticeable that fewer errors were made in the group with straight instruments (3.42 ± 3.45 vs. 7.5 ± 2.58, p = 0.005) (Fig. 5).

### 2D vs. 3D laparoscopy when using complex curved instruments

**Fig. 6:**
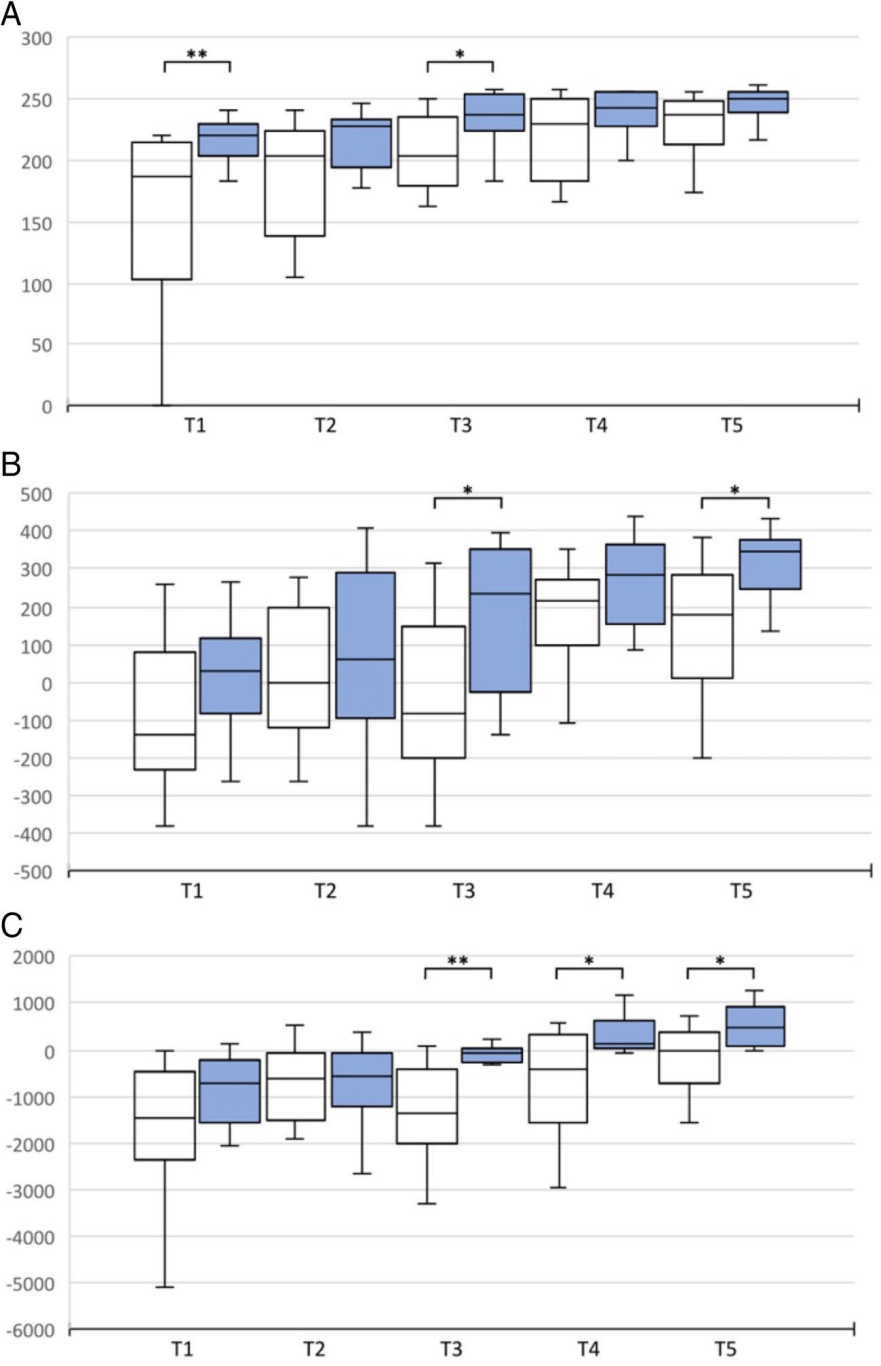
Performance score of PEG Transfer, Intracorporeal Suture and Transfer Task with curved instruments 2D vs. 3D visualization at T1-5. (**A**) PEG Transfer. **B** Intracorporeal Suture. **C** Transfer task. White: 2D visualization. Blue: 3D visualization. *p < 0.05; **p < 0.01

**Fig. 7:**
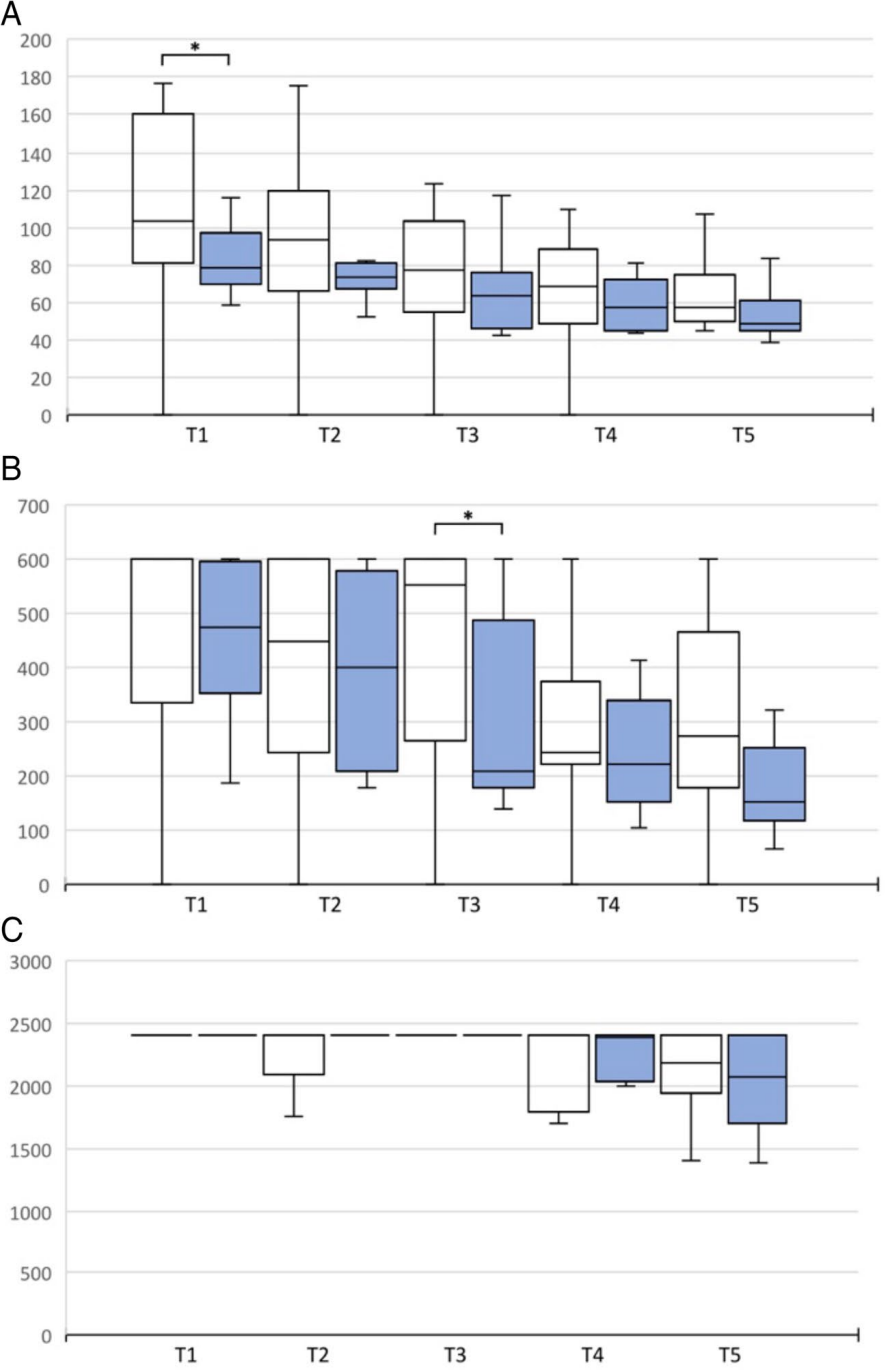
Time of PEG Transfer, Intracorporeal Suture and Transfer Task with curved instruments 2D vs. 3D visualization at T1-5. (**A**) PEG Transfer. **B** Intracorporal Suture. **C** Transfer task. White: 2D visualization. Blue: 3D visualization. *p < 0.05

In the PEG transfer exercise, subjects in the 3D group tended to perform better, although the difference was significant only at test time T1 (169.5 ± 54.1 vs. 216.6 ± 17.8) and T3 (208.3 ± 28.6 vs. 232.2 ± 24.6) (p = 0.01 and p = 0.033) (Fig. 6). The same was seen for the time required, but with only significance at test time T1 (118.8 ± 39.4 vs. 83.4 ± 17.8, p = 0.032) (Fig. 7). For the errors in the PEG Transfer and in the Pattern Cut exercise there were no differences. In the Intracorporeal Suture exercise, there was a significant advantage in the 3D group in performance (T5: 147.3 ± 191.5 vs. 294.4 ± 153.3, p = 0.017). Regarding the transfer exercise, a better performance was observed in the 3D group (T5: -251.7 ± 732.5 vs. 286.3 ± 1047.9, p = 0.024) (Fig. 6) with no difference in time (Fig. 7). For the reasons mentioned above, the errors were again examined independently. Here, the 3D group showed significantly fewer errors (T5: 7.5 ± 2.58 vs. 3.58 ± 2.94, p = 0.002).

## Discussion

The aim of the study was to examine the influence of 2D and 3D laparoscopy with straight and complex curved instruments on the performance of novices by means of four exercise tasks. For this purpose, analogous to the FLS curriculum, the exercises PEG Transfer, Pattern Cut and Intracorporeal Suture [21] were completed by the participants. To increase the complexity, a new transfer task was developed based in the three previous ones.

We were able to show that novices with conventional 2D visualization showed better performance with fewer errors as well as shorter time when performing the tasks with straight instruments compared to complex curved instruments. Especially in the PEG Transfer and Intracorporeal Suture exercises, the straight instrument group showed better performance. Martinec et al. also found poorer performance when using complex instruments [24]. However, the difference was significant only for PEG Transfer. Bensignor et al. studied a robotic needle holder compared with conventional laparoscopic instruments. Again, the complex instrument was found to be inferior for PEG Transfer, but with advantages for difficult sutures [25]. PEG Transfer is an exercise that trains depth perception and ambidextrous working in particular. In contrast, intracorporeal suturing requires significantly higher dexterity and a high degree of spatial orientation. It is therefore not surprising that curved instruments, which do not represent a straight-line extension of the arm, appear to be a hindrance during these exercises with a 2D visualization. Overall, the performance with the curved instruments show more varied results. This could be due to individual dexterity and adaptability. However, this circumstance was not investigated in this study, which can be seen as a limitation. The situation is different when cutting something out, as in the Pattern Cut exercise. This exercise is relatively static. The object to be cut is fixed to the surface. As a round shape has to be cut out, curved scissors could be advantageous in completing the task. Sieber et al. showed less deviation when cutting out a shape with a complex robotic instrument [15]. We found no difference in the two groups despite the more difficult two-dimensional perception and the complex curved instruments.

We could show in this study that 3D visualization is beneficial when using complex curved instruments. Especially in exercises where depth perception is essential (PEG Transfer and Intracorporeal Suture), the difficulty of using curved instruments in combination with conventional 2D visualization becomes apparent. We were also able to show advantages with 3D visualization in the transfer task. For the use of instruments with many degrees of freedom, which are not only an extension of the hand-arm axis of the surgeon, orientation seems to be facilitated by three-dimensionality [24]. This seems to be true not only for complex instruments but also for robotic-assisted surgery (RAS). In a comparison of a two-dimensional display in surgical robots with conventional laparoscopy, there was no advantage for RAS [26]. However, this is not surprising, as here the trial participants were skilled in conventional 2D laparoscopy. A similar study by Nio et al. showed an advantage in three-dimensional imaging in novices [27]. Although these studies were conducted some time ago, they highlight the need for 3D visualization in robotic systems. LaGrange et al. studied three different modalities in minimally invasive surgery: 3D robotic vs. 3D laparoscopic vs. 2D laparoscopic [28]. They emphasized that for more difficult tasks, 3D visualization was advantageous, especially when the aspect of increased degrees of freedom was included. The same seems to be true for the use of articulating laparoscopic instruments. Another group of researchers found that novices would benefit from 3D optics here [29]. They also found that especially in novices, complex articulating instruments should be combined with conventional straight instruments, as also performed in the study presented here. Bittner et al. investigated the use of an articulating needle holder under 2D and 3D vision [30]. They showed that more time was required to complete the exercises regardless of the visualization technique. Even though our study did not show a time advantage in the 3D group, we were able to highlight better performance. This is especially because time is only one aspect of the evaluation. Looking at the errors in independently, there are advantages in using 3D visualization when complex instruments are used.

Complex laparoscopic instruments have been developed primarily for single-port laparoscopy. A few studies can be found investigating their use in a conventional multi-port laparoscopy setting. Thus, comparison with other studies must be viewed critically. Since the subjects were participants without prior laparoscopic experience and had a relatively short training time of approximately 25 h, it must be assumed that the individual learning plateau of each participant had not yet been reached. This may lead to a bias in the results, even though according to Sroka et al. a significant improvement in performance was achieved after only 7.5 h of training on the simulator [31]. It also seems possible that the training modalities, in which each participant was able to complete as many training attempts as possible in a given time, led to individual differences in learning progress. Even if the prior stratification of the participants and the testing directly after the training phase minimize this source of error, documentation of the results and number of tasks performed per training unit could have provided information on this. As some authors call for more complex and clinically relevant exercises [32], we developed the described transfer task. However, this has not been validated and requires further investigation. For this reason, it was combined with common FLS tasks. Another weakness of the study is certainly the low case number of subjects. To overcome this problem and to compensate for the individual giftedness of the participants, a stratified randomization of the participants was performed. However, to further minimize this bias, a higher case number is necessary, as also emphasized by Zundel et al. [12]. In addition, it must be mentiones that, even though the assessment of the task was carried out strictly in accordance with the FLS curriculum, there was no blinded assessment of the participants. Even if the assessment was standardized, it was only carried out by one rater, which is a limitation overall.

## Conclusion

In our study we could show that learning laparoscopic techniques with complex curved instruments is more difficult with conventional standard 2D visualization. This is especially true for exercises with the need for adequate depth perception. However, this can be overcome using 3D optics. In addition, the use of curved instruments under three-dimensional vision seems to be advantageous when working on tasks with a higher degree of difficulty. This needs to be verified on a larger cohort and in the transfer to a real surgical situation.

### Supplementary Information

Below is the link to the electronic supplementary material.

Supplementary file1 (PDF 55 KB)

Supplementary file2 (PDF 47 KB)

Supplementary file3 (PDF 55 KB)

Supplementary file4 (PDF 44 KB)

Supplementary file5 (PDF 56 KB)

Supplementary file6 (PDF 45 KB)

Supplementary file7 (PDF 52 KB)

Supplementary file8 (PDF 43 KB)

Supplementary file9 (PDF 53 KB)

Supplementary file10 (PDF 42 KB)

Supplementary file11 (PDF 53 KB)

Supplementary file12 (PDF 42 KB)

## Data Availability

All data generated or analyzed during this study, including all used material, are included in this published article and its supplementary information files.

## Abbreviations

*FLS*: Fundamentals of laparoscopic surgery
*vs*: Versus
*MIS*: Minimally invasive surgery
*sec*: Seconds
*RAS*: Robotic assisted surgery
