# Supplementary material for: Laparoscopy training of novices with complex curved instruments using 2D- and 3D-visualization
Source: Langenbecks Arch Surg. 2024 Apr 3;409(1):109. doi: 10.1007/s00423-024-03297-w (PMC10990991; doi:10.1007/s00423-024-03297-w)
Supplement: Supplementary file 1 — Supplementary file1 (PDF 55 KB) [file 423_2024_3297_MOESM1_ESM.pdf]

Supplement 1.a. Performance score, procedure time and number of errors of PEG Transfer at test time T1-T5

	P-Score				Time (sec)				Errors (n)			
**Test Time**	**Group I** Mean ± SD (Range; Median)	**Group II** Mean ± SD (Range; Median)	**Group III** Mean ± SD (Range; Median)	**Group IV** Mean ± SD (Range; Median)	**Group I** Mean ± SD (Range; Median	**Group II** Mean ± SD (Range; Median)	**Group III** Mean ± SD (Range; Median)	**Group IV** Mean ± SD (Range; Median)	**Group I** Mean ± SD (Range; Median)	**Group II** Mean ± SD (Range; Median)	**Group III** Mean ± SD (Range; Median)	**Group IV** Mean ± SD (Range; Median)
T1	186.2±38.9 (117-236; 195)	169.5±54.1 (63-220; 191)	207.7±32 (115-233; 218)	216.6±17.8 (184-241; 221)	102.2±24.6 (64-143; 101)	118.8±39.4 (76-177)	92.3±32 (67-185; 82)	83.4±17.8 (59-116; 79)	0.58± 1 (0-3; 0)	0.42±0.67 (0-2; 0)	0±0 (0-0; 0)	0±0 (0-0; 0)
T2	224.1±8.6 (209-239; 225)	190.1±54.1 (104-220; 204.5)	223.3±21 (172-260; 227)	212.8±38.8 (108-247; 227)	74.3±7.3 (61-87; 74.5)	98.3±34.5 (59-175; 94.5)	70.1±16.5 (40-108; 70)	82.3±32.1 (53-172; 73)	0.08±0.29 (0-1; 0)	0.58±1.17 (0-4; 0)	0.33±0.49 (0-1; 0)	0.25±0.62 (0-2; 0)
T3	233.4±21.1 (179-255; 240)	208.3±28.6 (163-251; 210.5)	234.1±16.1 (206-257; 237.5)	232.2±24.6 (183-257; 237)	61.6±10.9 (45-81; 60)	81.8±25.5 (48-123; 79.5)	60.9±10.6 (43-77; 58)	66.2±21.9 (43-117; 63)	0.08±0.29 (0-1; 0)	0.5±0.67 (0-2; 0)	0.25±0.45 (0-1; 0)	0.08±0.29 (0-1; 0)
T4	239.9±14.4 (214-258; 245)	222.3±31.1 (166-258; 230.5)	240.9±9.9 (223-252; 241.5)	239.4±17.7 (199-256; 243)	56.8±9.1 (42-73; 55)	56.8±9.1 (42-73; 55)	56.8±9.1 (42-73; 55)	56.8±9.1 (42-73; 55)	0.17±0.39 (0-1; 0)	0.17±0.58 (0-2; 0)	0.17±0.39 (0-1; 0)	0.8±0.29 (0-1; 0)
T5	244.8±16.6 (196-258; 250.5)	232±23.6 (173-255; 240)	247.1±13.1 (217-262; 249.5)	246.3±12.9 (217-261; 251)	56.8±9.1 (42-73; 55)	56.8±9.1 (42-73; 55)	56.8±9.1 (42-73; 55)	56.8±9.1 (42-73; 55)	0.08±0.29 (0-1; 0)	0.17±0.39 (0-1; 0)	0.08±0.29 (0-1; 0)	0±0 (0-0; 0)

Group I: 2D visualization with straight instruments. Group II: 2D visualization with curved instruments. Group III: 3D visualization with straight instruments. Group IV: 3D visualization with curved instruments. SD: Standard deviation. P-Score: Performance score. Sec: Seconds.
