# Supplementary material for: Laparoscopy training of novices with complex curved instruments using 2D- and 3D-visualization
Source: Langenbecks Arch Surg. 2024 Apr 3;409(1):109. doi: 10.1007/s00423-024-03297-w (PMC10990991; doi:10.1007/s00423-024-03297-w)
Supplement: Supplementary file 2 — Supplementary file2 (PDF 47 KB) [file 423_2024_3297_MOESM2_ESM.pdf]

Supplement 1.b. Comparison of the different groups in terms of performance score, procedure time and number of errors of PEG Transfer at test time T1-T5.

	**P-Score**		**Time**		**Errors**	
**Test Time**	**Group I vs. Group II** (p-value)	**Group II vs. Group IV** (p-value)	**Group I vs. Group II** (p-value)	**Group II vs. Group IV** (p-value)	**Group I vs. Group II** (p-value)	**Group II vs. Group IV** (p-value)
T1	0.551	**0.01**	1	**0.032**	0.887	0.178
T2	**0.024**	0.114	0.146	0.763	0.291	0.5143
T3	**0.028**	**0.033**	0.062	0.267	0.16	0.16
T4	0.242	0.198	0.099	0.241	0.799	0.977
T5	0.06	0.068	**0.041**	0.229	0.755	0.514

For P-Score and number of errors Mann-Whitney-U-Test was used. For procedure time one-way ANOVA was used. Group I: 2D visualization with straight instruments. Group II: 2D visualization with curved instruments. Group IV: 3D visualization with curved instruments. Significance level was set at $p<0.05$ and highlighted bold. P-Score: Performance score.
