# Supplementary material for: Laparoscopy training of novices with complex curved instruments using 2D- and 3D-visualization
Source: Langenbecks Arch Surg. 2024 Apr 3;409(1):109. doi: 10.1007/s00423-024-03297-w (PMC10990991; doi:10.1007/s00423-024-03297-w)
Supplement: Supplementary file 3 — Supplementary file3 (PDF 55 KB) [file 423_2024_3297_MOESM3_ESM.pdf]

Supplement 2.a. Performance score, procedure time and number of errors of Pattern Cut at test time T1-T5.

Test Time	P-Score				Time (sec)				Errors (n)			
	Group I Mean ± SD (Range; Median)	Group II Mean ± SD (Range; Median)	Group III Mean ± SD (Range; Median)	Group IV Mean ± SD (Range; Median)	Group I Mean ± SD (Range; Median)	Group II Mean ± SD (Range; Median)	Group III Mean ± SD (Range; Median)	Group IV Mean ± SD (Range; Median)	Group I Mean ± SD (Range; Median)	Group II Mean ± SD (Range; Median)	Group III Mean ± SD (Range; Median)	Group IV Mean ± SD (Range; Median)
T1	-28.3±56.7 (-131-109; -30)	-26.8±47.8 (-100-81; -30)	10.3±65.1 (-100-107; -10)	-8.5±63.7 (-100-150; -4)	288.3±37 (171-300; 300)	283.5±46.7 (139-300; 300)	258±49.3 (178-300; 278)	270.2±50.3 (150-300; 300)	2±1.95 (0-7;1.5)	2.17±1.7 (0-5; 2)	1.58±1.56 (0-5; 1)	1.92±2.47 (0-8; 1)
T2	32.6±70.9 (-80-154; 29)	14.8±84.6 (-100-204; 0)	48.2±48.4 (-19-130; 51)	24.8±110.6 (-220-174; 19.5)	235.8±62.1 (126-300; 249)	253.6±75.6 (96-300; 300)	230.2±47.8 (164-299; 239.5)	225.2±74.9 (106-300; 226.5)	1.58±1.17 (0-4; 1.5)	1.58±1.88 (0-5; 1)	1.08±1.17 (0-4; 1)	2.5±3.5 (0-11; 1)
T3	63.6±64.7 (-38-166; 64)	54.7±100.1 (-96-210; 42)	109.1±58.3 (0-188; 126.5)	51.6±75.4 (-80-158; 65.5)	216.4±64.2 (134-300; 217)	223.7±79.3 (90-300; 248.5)	167.6±50 (112-300; 155)	208.4±53.9 (142-300; 210.5)	1±1.13 (0-3; 0.5)	1.08±1.56 (0-5; 0.5)	1.17±1.8 (0-5; 0)	2±2.3 (0-8; 1)
T4	68.3±69.4 (-120-157; 85.5)	66±86.4 (-79-185; 87.5)	121.6±43.9 (25-189; 124.5)	75.8±90.8 (-100-194; 84.5)	203.3±52.8 (103-300; 201)	199±79.3 (95-300; 177)	156.8±45.9 (111-275; 146)	187.5±59.4 (106-300; 190)	1.42±1.62 (0-6; 1)	1.75±1.6 (0-5; 1)	1.08±0.79 (0-2; 1)	1.83±2.69 (0-9; 1)
T5	106.5±76.8 (-33-208; 109)	105.5±62.6 (6-184; 90)	134.2±45.6 (52-205; 134)	119.7±70.4 (2-219; 128)	181.8±67.3 (91-267; 191)	169.5±60.5 (76-254; 171)	135.8±29 (95-198; 136)	157±55 (81-238; 158.5)	0.58±1.17 (0-4; 0)	1.25±0.97 (0-3; 1)	1.5±1.38 (0-4; 1.5)	1.17±1.4 (0-4; 1)

Group I: 2D visualization with straight instruments. Group II: 2D visualization with curved instruments. Group III: 3D visualization with straight instruments. Group IV: 3D visualization with curved instruments. SD: Standard deviation. P-Score: Performance score. Sec: Seconds.
