# Supplementary material for: Laparoscopy training of novices with complex curved instruments using 2D- and 3D-visualization
Source: Langenbecks Arch Surg. 2024 Apr 3;409(1):109. doi: 10.1007/s00423-024-03297-w (PMC10990991; doi:10.1007/s00423-024-03297-w)
Supplement: Supplementary file 4 — Supplementary file4 (PDF 44 KB) [file 423_2024_3297_MOESM4_ESM.pdf]

Supplement 2.b. Comparison of the different groups in terms of performance score, procedure time and number of errors of Pattern Cut at test time T1-T5.

	P-Score		Time		Errors	
**Test Time**	**Group I vs. Group II** (p-value)	**Group II vs. Group IV** (p-value)	**Group I vs. Group II** (p-value)	**Group II vs. Group IV** (p-value)	**Group I vs. Group II** (p-value)	**Group II vs. Group IV** (p-value)
T1	0.932	0.41	1	1	0.63	0.478
T2	0.347	0.671	1	1	0.63	0.671
T3	0.755	0.977	1	1	0.932	0.242
T4	0.932	0.887	1	1	0.671	0.59
T5	0.843	0.713	1	1	0.06	0.59

For P-Score and number of errors Mann-Whitney-U-Test was used. For procedure time one-way ANOVA was used. Group I: 2D visualization with straight instruments. Group II: 2D visualization with curved instruments. Group IV: 3D visualization with curved instruments. Significance level was set at $p<0.05$ and highlighted bold. P-Score: Performance score.
