# Supplementary material for: Laparoscopy training of novices with complex curved instruments using 2D- and 3D-visualization
Source: Langenbecks Arch Surg. 2024 Apr 3;409(1):109. doi: 10.1007/s00423-024-03297-w (PMC10990991; doi:10.1007/s00423-024-03297-w)
Supplement: Supplementary file 5 — Supplementary file5 (PDF 56 KB) [file 423_2024_3297_MOESM5_ESM.pdf]

Supplement 3.a. Performance score, procedure time and number of errors of Intracorporeal Suture at test time T1-T5.

	P-Score				Time (sec)				Errors (n)			
Test Time	Group I Mean ± SD (Range; Median)	Group II Mean ± SD (Range; Median)	Group III Mean ± SD (Range; Median)	Group IV Mean ± SD (Range; Median)	Group I Mean ± SD (Range; Median)	Group II Mean ± SD (Range; Median)	Group III Mean ± SD (Range; Median)	Group IV Mean ± SD (Range; Median)	Group I Mean ± SD (Range; Median)	Group II Mean ± SD (Range; Median)	Group III Mean ± SD (Range; Median)	Group IV Mean ± SD (Range; Median)
T1	80.2±151.5 (-154-331; 143)	-98.1 ±204.6 (-380-258; -180)	99±142.3 (-120-324; 91)	1±179.3 (-380-264.5; 26.8)	451.7±122 (264-600; 407.5)	521.3 ±134.9 (242-600; 600)	411.4 ±159.9 (176-600; 407.5)	460.7 ±135.5 (188-600; 477)	0.34±0.9 (0-3; 0)	1.58±1.39 (0-4; 2)	1.46±2.39 (0-8; 0.5)	2.2±3.47 (0-11; 0.88)
T2	207.8±93.7 (29-350; 194)	19.3±180.1 (-260-279; 3.3)	242.1 ±110.4 (44-405.5; 267.5)	70.4±228.3 (-380-409; 58.5)	294.9±99.3 (150-471; 302)	458±161 (221-600; 521.5)	262.5±97.9 (154-446; 246.5)	393.6 ±165.8 (181-600; 401.5)	0.32±0.78 (0-2.5; 0)	1.94±2.63 (0-6.5; 1)	1.21±1.6 (0-4.5; 0.63)	1.23±1.85 (0-6; 0.63)
T3	257.1±95 (63-396; 278)	-38.2 ±237.2 (-380-317; -92)	321.8±43.3 (277.5-422; 304.8)	172.8 ±203.8 (-140-398; 234)	238.2±94.2 (104-427; 217.5)	468.4 ±166.6 (173-600; 576)	179.1±44.6 (78-240; 188)	314.9 ±175.4 (142-600; 208.5)	0.5±0.67 (0-2; 0)	0.86±1.26 (0-3; 0.25)	0.74±1.27 (0-4.25; 0)	2.68±4.86 (0-16; 1.13)
T4	255.9 ±113.6 (-4-419.5; 232.5)	189.6 ±124.4 (-105-353.5; 236)	218.7 ±172.1 (-38-521; 316.5)	269±116.5 (83-441; 282.5)	249.3 ±103.8 (123-504; 250.5)	305.8 ±121.5 (144-600; 264)	224.6 ±153.8 (79-538; 166.5)	239.6 ±106.1 (105-412; 220.5)	0.31±0.9 (0-3; 0)	0.53±0.99 (0-3; 0)	1.04±1.32 (0-4; 0.25)	0.83±1.6 (0-5; 0)
T5	305±100.5 (7-401; 32.,8)	147.3 ±191.5 (-202.5-384; 202.5)	319.7 ±149.8 (-15-473; 380)	294.4 ±153.3 (-119-435; 348)	188.1±82.7 (99-423; 177)	329.8 ±165.4 (116-600; 287.5)	185.1 ±137.5 (65-505; 123.5)	198.5 ±145.8 (65-599; 152)	0.75±2.09 (0-7; 0)	0.97±1,03 (0-2.75; 0.5)	0.35±0.96 (0-3.25; 0)	0.65±1.33 (0-4; 0)

Group I: 2D visualization with straight instruments. Group II: 2D visualization with curved instruments. Group III: 3D visualization with straight instruments. Group IV: 3D visualization with curved instruments. SD: Standard deviation. P-Score: Performance score. Sec: Seconds.
