# Supplementary material for: Laparoscopy training of novices with complex curved instruments using 2D- and 3D-visualization
Source: Langenbecks Arch Surg. 2024 Apr 3;409(1):109. doi: 10.1007/s00423-024-03297-w (PMC10990991; doi:10.1007/s00423-024-03297-w)
Supplement: Supplementary file 6 — Supplementary file6 (PDF 45 KB) [file 423_2024_3297_MOESM6_ESM.pdf]

Supplement 3.b. Comparison of the different groups in terms of performance score, procedure time and number of errors of Intracorporeal Suture at test time T1-T5.

	P-Score		Time		Errors	
**Test Time**	**Group I vs. Group II** (p-value)	**Group II vs. Group IV** (p-value)	**Group I vs. Group II** (p-value)	**Group II vs. Group IV** (p-value)	**Group I vs. Group II** (p-value)	**Group II vs. Group IV** (p-value)
T1	**0.028**	0.266	1	1	**0.008**	0.652
T2	**0.007**	0.514	**0.03**	1	0.06	0.566
T3	**0.002**	**0.028**	**0.001**	**0.039**	0.722	0.159
T4	0.291	0.114	1	1	0.319	0.671
T5	**0.012**	**0.017**	0.087	0.137	**0.033**	0.142

For P-Score and number of errors Mann-Whitney-U-Test was used. For procedure time one-way ANOVA was used. Group I: 2D visualization with straight instruments. Group II: 2D visualization with curved instruments. Group IV: 3D visualization with curved instruments. Significance level was set at $p<0.05$ and highlighted bold. P-Score: Performance score.
