# Supplementary material for: Laparoscopy training of novices with complex curved instruments using 2D- and 3D-visualization
Source: Langenbecks Arch Surg. 2024 Apr 3;409(1):109. doi: 10.1007/s00423-024-03297-w (PMC10990991; doi:10.1007/s00423-024-03297-w)
Supplement: Supplementary file 7 — Supplementary file7 (PDF 52 KB) [file 423_2024_3297_MOESM7_ESM.pdf]

Supplement 4.a. Performance score and procedure time of transfer task at test time T1-T5.

Test Time	P-Score				Time (sec)			
	Group I<br>Mean ± SD<br>(Range; Median)	Group II<br>Mean ± SD<br>(Range; Median)	Group III<br>Mean ± SD<br>(Range; Median)	Group IV<br>Mean ± SD<br>(Range; Median)	Group I<br>Mean ± SD<br>(Range; Median)	Group II<br>Mean ± SD<br>(Range; Median)	Group III<br>Mean ± SD<br>(Range; Median)	Group IV<br>Mean ± SD<br>(Range; Median)
T1	-3610.3±4092.7 (-14854-394.5; -2595.8)	-1840.5±1452 (-5075-151; -1646.5)	-1260±1322.7 (-4870.5-136; -856.5)	-1215.5±1613.3 (-5904-119; -737)	2385±52 (2220-2400; 2400)	2379.6±70.7 (2155-2400; 2400)	2400±0 (2400-2400; 2400)	2387.2±44.5 (2246-2400; 2400)
T2	-1206.9±1947.1 (-6624-780; -626.5)	-986.4±1382.4 (-4638-534; -734)	-780.9±827.6 (-2988-75; -726)	-700.2±878.5 (-2670.5-395; -550)	2339.9±167.5 (1825-2400; 2400)	2274.7±296.9 (1531-2400; 2400)	2349.6±141.5 (1909-2400; 2400)	2366.2±84.5 (2127-2400; 2400)
T3	-652.8±1259.6 (-3652.5-1238; -263)	-1424.8±1014.7 (-3329.5-86; -1415.5)	-766.2±1031.4 (-2758-785.5; -963.3)	-208.3±467.3 (-1594-245; -93.8)	2301.8±286 (1410-2400; 2400)	2400±0 (2400-2400; 2400)	2311±205.6 (1847-2400; 2400)	2400±0 (2400-2400; 2400)
T4	-435.7±848.7 (-2104-1201.5; -486)	-769.1±1181.4 (-2961-566; -658.5)	-383.6±1183.8 (-3849-502.5; 81)	184.5±621.6 (-1398.5-1184; 147)	2279.4±270.7 (1494-2400; 2400)	2202.3±295.7 (1702-2400; 2400)	2254.8±246.9 (1741-2400; 2400)	2172±361.8 (1422-2400; 2400)
T5	-186.4±1018.1 (-2468-1227; -229.5)	-251.7±732.5 (-1581-746; -222.5)	-161.6±805.2 (-2237.5-1112; -134.5)	286.3±1047.9 (-2777-1272.5; 464.5)	2209.5±350.9 (1435-2400; 2400)	2173.8±306.6 (1401-2400; 2290.5)	2259.3±235.1 (1578-2400; 2343.5)	2035.3±391.5 (1376-2400; 2064)

Group I: 2D visualization with straight instruments. Group II: 2D visualization with curved instruments. Group III: 3D visualization with straight instruments. Group IV: 3D visualization with curved instruments. SD: Standard deviation. P-Score: Performance score. Sec: Seconds.
