# Supplementary material for: Laparoscopy training of novices with complex curved instruments using 2D- and 3D-visualization
Source: Langenbecks Arch Surg. 2024 Apr 3;409(1):109. doi: 10.1007/s00423-024-03297-w (PMC10990991; doi:10.1007/s00423-024-03297-w)
Supplement: Supplementary file 8 — Supplementary file8 (PDF 43 KB) [file 423_2024_3297_MOESM8_ESM.pdf]

Supplement 4.b. Comparison of the different groups in terms of performance score and procedure time of transfer task at test time T1-T5.

	P-Score		Time	
**Test Time**	**Group I vs. Group II** (p-value)	**Group II vs. Group IV** (p-value)	**Group I vs. Group II** (p-value)	**Group II vs. Group IV** (p-value)
T1	0.291	0.128	1	1
T2	0.932	0.731	1	1
T3	0.078	0.006	1	1
T4	0.843	0.039	1	1
T5	0.713	0.024	1	1

For P-Score Mann-Whitney-U-Test was used. For procedure time one-way ANOVA was used. Group I: 2D visualization with straight instruments. Group II: 2D visualization with curved instruments. Group IV: 3D visualization with curved instruments. Significance level was set at $p<0.05$ and highlighted bold. P-Score: Performance score.
