# Supplementary material for: Laparoscopy training of novices with complex curved instruments using 2D- and 3D-visualization
Source: Langenbecks Arch Surg. 2024 Apr 3;409(1):109. doi: 10.1007/s00423-024-03297-w (PMC10990991; doi:10.1007/s00423-024-03297-w)
Supplement: Supplementary file 9 — Supplementary file9 (PDF 53 KB) [file 423_2024_3297_MOESM9_ESM.pdf]

Supplement 5.a. Number of errors and sum of miscuts of transfer task at test time T1-T5.

Test Time	Errors (n) Group I Mean ± SD (Range; Median)	Errors (n) Group II Mean ± SD (Range; Median)	Errors (n) Group III Mean ± SD (Range; Median)	Errors (n) Group IV Mean ± SD (Range; Median)	Sum of miscuts (mm) Group I Mean ± SD (Range; Median)	Sum of miscuts (mm) Group II Mean ± SD (Range; Median)	Sum of miscuts (mm) Group III Mean ± SD (Range; Median)	Sum of miscuts (mm) Group IV Mean ± SD (Range; Median)
T1	-14.67±8.95 (3-32; 13)	9.75±6.59 (1-22; 8)	8.67±6.79 (2-28; 7)	-8.42±5.47 (2-21; 7.5)	164.33±141.11 (45-470; 109)	70.08±84.76 (2-241; 25.5)	64.58±85.11 (5-247; 22.5)	91.5±71.31 (10-204; 67.5)
T2	9.42±4.74 (3-20; 7.5)	7.08±4.66 (0-15; 5.5)	8.08±4.85 (1-18; 7.5)	6.33±3.55 (1-13; 6)	100±87.73 (18-275; 61.5)	55.25±36.28 (0-123; 51)	70.17±73.73 (3-192; 30)	63.42±79.6 (5-266; 33.5)
T3	9±4.71 (4-21; 7.5)	7.75±6.61 (1-21; 4.5)	7.42±3.73 (3-16; 6.5)	5.17±2.17 (2-10; 5)	96.83±119.62 (15-400; 45)	64.75±92.14 (3-323; 26)	38.83±33.09 (2-107; 30)	42.33±53.21 (2-186 ± 18.5)
T4	6.75±3.25 (1-12; 6.5)	6.17±5.59 (1-20; 4.5)	5.58±4.91 (0-19; 5)	5.42±2.94 (2-11; 5)	90.5±96.19 (3-339; 52)	41.96±50.5 (0-151; 21,75)	32.17±36.22 (0-110; 18.5)	47.42±39.64 (11-149; 35.5)
T5	7±3.95 (1-14; 6)	5.58±3.53 (0-14; 5)	4.83±5.34 (1-21; 3)	4.08±3.15 (0-11; 3.5)	79.33±103.71 (2-364; 32.5)	45.83±54.3 (0-199; 31)	42.25±49.93 (2-127; 12)	26.58±38.91 (0-140; 13.5)

Miscuts over the tolerance range was counted as an error (number of errors). In addition, miscuts that ran over a longer distance were measured (sum of miscuts). Group I: 2D visualization with straight instruments. Group II: 2D visualization with curved instruments. Group III: 3D visualization with straight instruments. Group IV: 3D visualization with curved instruments. SD: Standard deviation.
