# Supplementary material for: Laparoscopy training of novices with complex curved instruments using 2D- and 3D-visualization
Source: Langenbecks Arch Surg. 2024 Apr 3;409(1):109. doi: 10.1007/s00423-024-03297-w (PMC10990991; doi:10.1007/s00423-024-03297-w)
Supplement: Supplementary file 10 — Supplementary file10 (PDF 42 KB) [file 423_2024_3297_MOESM10_ESM.pdf]

Supplement 5.b. Comparison of the different groups in terms of number of errors and sum of miscuts of transfer task at test time T1-T5.

	Errors		Sum of miscuts	
**Test Time**	**Group I vs. Group II** (p-value)	**Group II vs. Group IV** (p-value)	**Group I vs. Group II** (p-value)	**Group II vs. Group IV** (p-value)
T1	0.178	0.713	**0.02**	0.319
T2	0.219	0.755	0.347	0.478
T3	0.266	0.843	0.219	0.63
T4	0.378	0.799	0.078	0.198
T5	0.443	0.242	0.671	0.178

Mann-Whitney-U-Test was used. Group I: 2D visualization with straight instruments. Group II: 2D visualization with curved instruments. Group IV: 3D visualization with curved instruments. Significance level was set at $p<0.05$ and highlighted bold.
