# Supplementary material for: Laparoscopy training of novices with complex curved instruments using 2D- and 3D-visualization
Source: Langenbecks Arch Surg. 2024 Apr 3;409(1):109. doi: 10.1007/s00423-024-03297-w (PMC10990991; doi:10.1007/s00423-024-03297-w)
Supplement: Supplementary file 11 — Supplementary file11 (PDF 53 KB) [file 423_2024_3297_MOESM11_ESM.pdf]

Supplement 6.a. Number of residuals and sum of residuals of transfer task at test time T1-T5.

	Residuals (n)				Sum of residuals (mm)			
Test Time	Group I<br>Mean ± SD<br>(Range; Median)	Group II<br>Mean ± SD<br>(Range; Median)	Group III<br>Mean ± SD<br>(Range; Median)	Group IV<br>Mean ± SD<br>(Range; Median)	Group I<br>Mean ± SD<br>(Range; Median)	Group II<br>Mean ± SD<br>(Range; Median)	Group III<br>Mean ± SD<br>(Range; Median)	Group IV<br>Mean ± SD<br>(Range; Median)
T1	8.5±6.3<br>(1-20; 8)	9.33±5.18<br>(4-22; 8.5)	7.25±4.65<br>(0-16; 8.5)	7±3.05<br>(3-14; 6.5)	166.08±273.38<br>(3-989; 94)	123.5±58.53<br>(22-227; 128.5)	100.67±137.32<br>(0-519; 67)	85.92±81.22<br>(9-256; 70.5)
T2	5.08±4.98<br>(0-14; 3.5)	9.25±4.62<br>(4-21; 8)	7.17±4.06<br>(2-13;8)	6.75±4.98<br>(0-18; 7)	88.67±94.1<br>(0-327; 81.5)	88.33±57.32<br>(8-216; 75)	61.25±48.82<br>(11-178; 57.5)	67.08±59.15<br>(0-190; 46.5)
T3	4.08±3.5<br>(0-10; 4)	8.83±3.76<br>(2-14; 9.5)	7.25±5.36<br>(1-14; 8.5)	5.42±2.97<br>(0-11; 6)	40.5±46.85<br>(0-138; 14.5)	112.75±70.73<br>(8-237; 117.5)	77.17±60.99<br>(3-159; 99.5)	41.83±29.62<br>(0-119; 36.5)
T4	4.67±3.6<br>(0-13; 4)	9.42±3.97<br>(4-17; 9)	6.33±4.14<br>(2-15; 5)	4.17±2.04<br>(1-8; 4)	38.17±35.5<br>(0-102; 25.5)	80.17±61.31<br>(5-215; 56)	54.83±51.68<br>(8-155; 29.5)	26.67±34.46<br>(2-129; 14)
T5	3.42±3.45<br>(0-11; 2.5)	7.5±2.58<br>(3-11; 7.5)	5.25±3.36<br>(0-11; 4.5)	3.58±2.94<br>(0-11; 3.5)	20±25.84<br>(0-78; 10)	57.75±35.54<br>(8-116; 48)	43.92±36.34<br>(0-120; 47.5)	37.67±52.16<br>(0-187; 25)

The red marked residual tissue was also evaluated as number (number of residuals) and distance value (sum of residuals). Group I: 2D visualization with straight instruments. Group II: 2D visualization with curved instruments. Group III: 3D visualization with straight instruments. Group IV: 3D visualization with curved instruments. SD: Standard deviation.
