# Supplementary material for: Laparoscopy training of novices with complex curved instruments using 2D- and 3D-visualization
Source: Langenbecks Arch Surg. 2024 Apr 3;409(1):109. doi: 10.1007/s00423-024-03297-w (PMC10990991; doi:10.1007/s00423-024-03297-w)
Supplement: Supplementary file 12 — Supplementary file12 (PDF 42 KB) [file 423_2024_3297_MOESM12_ESM.pdf]

Supplement 6.b. Comparison of the different groups in terms of number of residuals and sum of residuals of transfer task at test time T1-T5.

	Residuals		Sum of residuals	
**Test Time**	**Group I vs. Group II** (p-value)	**Group II vs. Group IV** (p-value)	**Group I vs. Group II** (p-value)	**Group II vs. Group IV** (p-value)
T1	0.63	0.378	0.319	0.089
T2	**0.045**	0.16	0.799	0.266
T3	**0.006**	**0.017**	**0.005**	**0.028**
T4	**0.005**	**0.0**	0.068	**0.002**
T5	**0.005**	**0.002**	**0.006**	**0.045**

Mann-Whitney-U-Test was used. Group I: 2D visualization with straight instruments. Group II: 2D visualization with curved instruments. Group IV: 3D visualization with curved instruments. Significance level was set at $p<0.05$ and highlighted bold.
